# Report on Dental Chemistry

**Published:** 1870-09

**Authors:** John Allen


### DENTAL REGISTER.

Vol. XXIV.] SEPTEMBER, 1870. [No. 9.

### Original Communications.

### REPORT ON DENTAL CHEMISTRY.

BY DR. JOHN ALLEN.

**Read before the American Dental Association.**

As organic chemistry treats of the substances which form the structure of organized bodies and their products whether animal or vegetable, let us look at dental chemistry a moment, from two stand points, viz.: the constituents of which the teeth are formed, and the source from which those constituents are derived.

According to Johnston's chemical analysis the constituents which enter into and are embodied in the osseous structure of the human teeth are as follows:

Phosphate of lime, with traces of fluoride of calcium, 66.72; carbonate of lime, 3.36; soluble salts, 0.83; cartilage, 27.61; fat, 0.40: phosphate of lime, 1.08.

The enamel, or vitreous substance which covers the crown of a human tooth is composed of phosphate of lime and traces of calcium, 87.82; carbonate of lime, 4.37; phosphate of magnesia, 1.34; soluble salts, 0.88; organic substances, 3.39; fat, 0.20.

These constituents form the basis of the bones and teeth of a person. In order, therefore, to produce a good osseous structure of the human frame such articles of food should be used as contain a due proportion of these constituents, for such are the operations of nature in the animal economy that when food is taken into the system it is duly apportioned and converted into muscle, bone, fat, etc. For this purpose the necessary materials of which our systems are formed exist in the proper nourishment designed for man. But by the present mode of preparing certain articles of food (some of which we will notice) a large portion of the essential elements for osseous formations are taken out and discarded. For example, the wheat, rye, etc., of which bread is formed, is stripped of the hull and coarser portions of the grain which are requisite materials for bones and teeth, as deduced from the following well authenticated chemical analysis, by which it is found that in 500 lbs. of whole grain there is :

Muscle material, 78 lbs.; of the fat principle, 12 lbs.; of the inorganic elements for bone, etc., 85 lbs. 500 lbs. of fine flour contains: muscle material, 65 lbs.; fat principle, 10 lbs.; bone material, 30 lbs. 500 lbs; of bran contains, muscle material, none; fat principle, 30 lbs.; bone material, 125 lbs.

The foregoing facts should teach the importance of using such food as contains the requisite elements for developing and sustaining a perfect organism in all its parts.

By close and careful scientific researches we have sufficient light upon this subject to serve as a guide which we should follow, for nature is so independent in the administration of her laws that those who disregard or depart from them must abide the result. Food for children ought to be plain and substantial; such as bread, milk, eggs, potatoes, rice, beans, etc. These constitute the principal articles of food necessary for a good development of the human system.

Adults require the same constituents to sustain the organization of the body that the young do to produce it; otherwise deterioration and decay ensue. More meat may be used as persons grow older.

Condiments possess none of the necessary elements for producing or sustaining a healthy organism.

Candies, which contain poisonous substances, (as many of them do), are detrimental. Many of the confectioners in coloring their candies, etc., employ the following materials: For their greens they use Brunswick green, carbonate of copper, or arsenite of copper. For their yellows, chromate of lead, or gamboge. For their reds, red lead, vermilion or cinnabar; and for their whites, white lead. All of which are poisonous. Although some confectioners use coloring ingredients comparatively harmless, such as saffron, French berries, Persian berries, fustic wood, etc., for yellows. For reds, cochineal, including carmine, Brazil wood, and madder. For blues, litmus and indigo. For greens, mixtures of any of the above vegetable yellow with indigo. If the eye *must* be gratified as well as the taste, in these matters, the latter colorings are far preferable to the former, but the purchaser of the candies can seldom tell the one from the other, therefore it is better to use them sparingly.

There are many articles of luxury used that are deleterious to the general health when indulged in too freely, that do not act directly upon the teeth, but indirectly prove injurious by producing vitiated secretions which affect them. Pure saccharine substances, when used moderately, are not considered injurious to the teeth, but when used in *excess* they become detrimental. When these are taken into the system they are converted into lactic acid. If this becomes predominant in the salivary secretions it attacks the lining portions of the teeth, and thus produces decay.

Now, as a profession we should do all in our power to diffuse practical information with reference to the best means of forming and preserving the natural teeth, for however perfect we may be able to construct artificial dentures, man can not do the work of his Creator as well as He can do it Himself.

